# Near-Infrared Fluorescence Tumor-Targeted Imaging in Lung Cancer: A Systematic Review

**DOI:** 10.3390/life12030446

**Published:** 2022-03-17

**Authors:** Lisanne K. A. Neijenhuis, Lysanne D. A. N. de Myunck, Okker D. Bijlstra, Peter J. K. Kuppen, Denise E. Hilling, Frank J. Borm, Danielle Cohen, J. Sven D. Mieog, Willem H. Steup, Jerry Braun, Jacobus Burggraaf, Alexander L. Vahrmeijer, Merlijn Hutteman

**Affiliations:** 1 Department of Surgery, Leiden University Medical Center, 2333 ZA Leiden, The Netherlands; l.k.a.neijenhuis@lumc.nl (L.K.A.N.); l.d.a.n.de_muynck@lumc.nl (L.D.A.N.d.M.); o.d.bijlstra@lumc.nl (O.D.B.); p.j.k.kuppen@lumc.nl (P.J.K.K.); d.e.hilling@lumc.nl (D.E.H.); j.s.d.mieog@lumc.nl (J.S.D.M.); a.l.vahrmeijer@lumc.nl (A.L.V.); 2 Centre for Human Drug Research, 2333 CL Leiden, The Netherlands; kb@chdr.nl; 3 Department of Surgery, Erasmus Medical Center, 3015 GD Rotterdam, The Netherlands; 4 Department of Pulmonology, Leiden University Medical Center, 2333 ZA Leiden, The Netherlands; f.j.borm@lumc.nl; 5 Department of Pathology, Leiden University Medical Center, 2333 ZA Leiden, The Netherlands; d.cohen@lumc.nl; 6 Department of Surgery, HAGA Hospital, 2545 AA The Hague, The Netherlands; w.steup@hagaziekenhuis.nl; 7 Department of Cardiothoracic Surgery, Leiden University Medical Center, 2333 ZA Leiden, The Netherlands; j.braun@lumc.nl

**Keywords:** lung cancer, near-infrared fluorescence imaging, fluorescence-guided surgery, molecular imaging, optical imaging

## Abstract

Lung cancer is the most common cancer type worldwide, with non-small cell lung cancer (NSCLC) being the most common subtype. Non-disseminated NSCLC is mainly treated with surgical resection. The intraoperative detection of lung cancer can be challenging, since small and deeply located pulmonary nodules can be invisible under white light. Due to the increasing use of minimally invasive surgical techniques, tactile information is often reduced. Therefore, several intraoperative imaging techniques have been tested to localize pulmonary nodules, of which near-infrared (NIR) fluorescence is an emerging modality. In this systematic review, the available literature on fluorescence imaging of lung cancers is presented, which shows that NIR fluorescence-guided lung surgery has the potential to identify the tumor during surgery, detect additional lesions and prevent tumor-positive resection margins.

## 1. Introduction

Lung cancer is the most common cancer type worldwide [1]. Two main histological classes of lung cancer exist: small-cell lung carcinomas (SCLCs) and non-small cell lung carcinomas (NSCLCs). Surgical resection is the cornerstone in the curative-intended treatment of non-disseminated NSCLCs [2]. The primary goal in surgical oncology treatment is to achieve a complete resection of the tumor (R0). Incomplete surgical resection of the tumor (R1 or R2) occurs in 2.8–12.7% of patients and is associated with significantly worse survival and higher recurrence rates [3,4,5,6]. In the past, lung resections were mostly performed via thoracotomy. To improve recovery and postoperative pain, there has been a shift from open to minimally invasive surgery (video-assisted thoracoscopic surgery and robotic-assisted thoracoscopic surgery) [7]. A limitation of these surgical techniques is reduced tactile feedback. With the advent of lung cancer screening programs, it is expected that in the near future, more resections will be performed for small nodules, which can be hard to identify during (minimally invasive) surgery [8]. These small-size pulmonary nodules can be operated on with lung-parenchyma-sparing operations (i.e., segmentectomy), with good clinical and oncological outcomes [9]. There is a need for improved preoperative and/or intraoperative imaging techniques to both localize lung nodules and guarantee R0 resections.

Various preoperative techniques facilitating image-guided localization of pulmonary nodules during surgical resection have been described. This includes several computed tomography (CT)-guided modalities, such as 3D planning, preoperative placement of hook wires, microcoils, fiducials, or radiotracers [10,11,12,13]. These techniques can help in localizing the lesion but commonly have insufficient resolution to determine resection margins. Moreover, interventional CT-guided methods carry a risk of complications, such as pneumothorax, hemorrhage, and air embolisms [14]. A safer technique to detect adequate resection margins intraoperatively is frozen section analysis, but this is an expensive, time-consuming procedure that requires a pathologist [15]. Frozen section analysis may result in false negatives since the outcome is dependent on the location where the sections are taken [16]. Intraoperative ultrasound has also been performed for the localization of pulmonary nodules, but this technique requires a specialized radiologist and is best performed in a totally collapsed lung, which is often not possible due to emphysema [17]. Therefore, there is an unmet need for a real-time, safe, and reliable intraoperative imaging technique.

**Figure 1:**
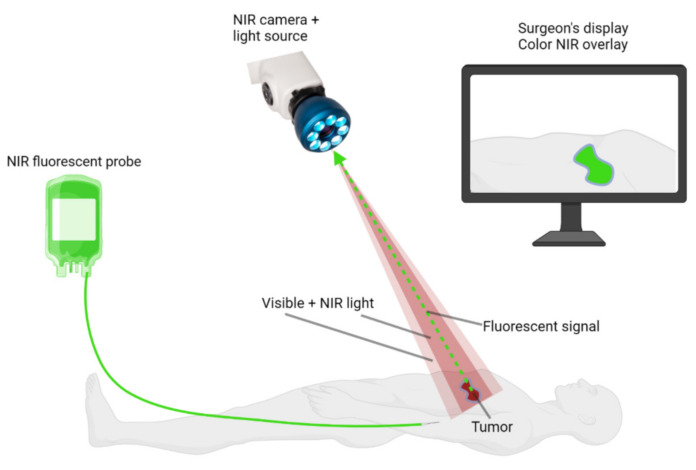
Basic principles of NIR fluorescence-guided surgery. *A NIR fluorescent probe is administered to the patient intravenously, intraparenchymally, or topically. A NIR fluorescence imaging system is used to visualize the probe during surgery. These systems require NIR excitation light, collection optics and filtration, and a camera sensitive to NIR fluorescence emission light. NIR fluorescence images are displayed on a screen, and these images are merged with white-light images of the surgical field. Created with BioRender.com*.

Intraoperative, near-infrared (NIR) fluorescence imaging has the potential to overcome several of these problems. NIR fluorescence imaging is a noninvasive real-time technique. NIR light has a wavelength ranging from 650 nm to 900 nm and is superior to visible light for intraoperative imaging due to its deeper penetration in tissue, up to 10 mm [18]. Since tissue shows only limited autofluorescence within the NIR spectrum, the contrast between the fluorescence signal in the tumor and healthy tissue can be maximized using NIR fluorescent probes. In addition, NIR light does not affect the surgical field, as NIR light is invisible to the human eye. NIR fluorescence-guided surgery requires a fluorophore, a light source, and a camera (Figure 1). The light source emits photons that will be absorbed by the fluorophore. This results in the excitation of the fluorophore by the photon. When the fluorophore returns to its ground state, it emits a photon of lower energy and higher wavelength. Both the excitation and emission photons are influenced by reflection and refraction when passing through different types of tissues, leading to scattering, which ultimately leads to blurred images [19]. The emitted photons can be detected with a camera and are displayed in real time on a monitor. The currently available imaging systems merge the white light illumination of the surgical field with the NIR fluorescence images, providing the surgeon with both anatomical information and fluorescence information simultaneously. A fluorescent probe is usually administered intravenously. There are two types of fluorescent probes: non-specific fluorophores or fluorophores conjugated to a targeting vehicle. At present, three non-specific fluorophores, indocyanine green (ICG), methylene blue (MB), and 5-aminolevulinic acid (5-ALA), are approved by the United States Food and Drug Administration (FDA) and the European Medicines Agency (EMA).

NIR fluorescence-guided surgery has the potential to visualize the tumor in real time and to distinguish between tumor and normal tissues. Therefore, it could be used to detect the surgical target, identify novel (small) malignant lesions, and prevent tumor-positive resection margins. Its potential utility has been studied in several types of cancers (e.g., hepatocellular carcinoma, colorectal cancer, ovarian cancer, breast cancer, and gliomas) [20,21,22,23,24]. This technique has also been studied for different surgical procedures for lung cancer. Several studies showed that NIR fluorescence-guided surgery could be useful for sentinel node identification [25,26,27]. Intraoperative imaging with fluorescence has also been used to successfully identify the intersegmental plane to perform more lung-preserving procedures, especially for smaller tumors [28,29,30]. Preoperative CT-guided transthoracic injection or injection via bronchoscopy of non-specific fluorophores the ICG and MB are able to detect most pulmonary nodules during surgery [31,32,33,34,35]. However, these techniques carry the risk of several complications and require prior information about the localization of the tumor. The use of NIR fluorescence-guided surgery as a single modality, whereby the fluorescent probe is administered to the patient via the peripheral route, is non-invasive, has limited risks, and does not require prior information about the localization of the tumor.

This review provides an overview of preclinical and clinical applications of tumor-targeted NIR fluorescence-guided surgery in lung cancer and their metastases with the use of either a non-specific fluorophore or fluorophore conjugated to a targeting vehicle.

## 2. Materials and Methods

A systematic search was conducted by the librarian of the Leiden University Medical Center in the PubMed, Cochrane, Web of Science, and Embase databases. Terms of the search strategy consisted of “lung cancer”, “pulmonary nodes”, “fluorescence imaging”, “near-infrared imaging”, “optical imaging”, and “intraoperative fluorescence imaging”. The search strategy for each database is shown in Appendix A. The last search was conducted on 29 September 2021. First, all articles were screened based on the title and abstract. Subsequently, full article screening was performed. The inclusion criteria were defined as intraoperative or in vivo detection of lung cancer/pulmonary nodes or metastases of lung cancer with near-infrared fluorescence imaging, and only English manuscripts were considered. Exclusion criteria were defined as reviews, case reports, conference abstract, no full text available, and multimodality imaging.

## 3. Results and Discussion

### 3.1. Literature Search

**Figure 2:**
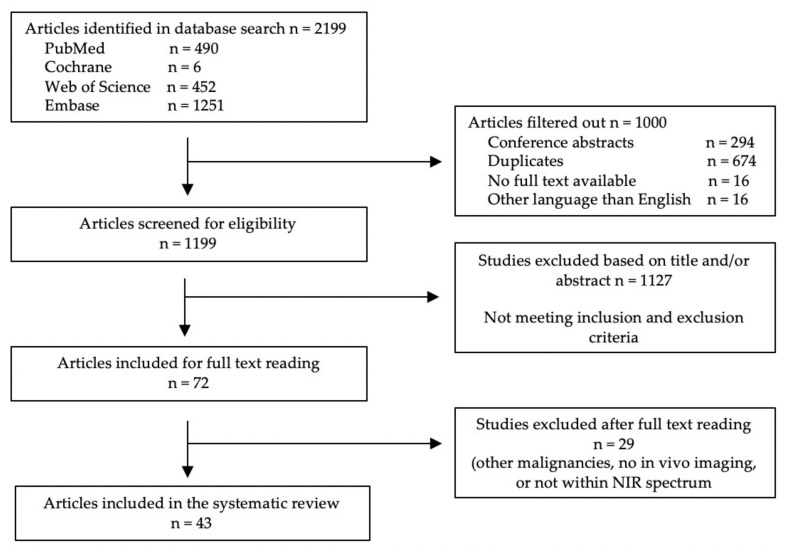
Summary of study selection.

A total of 2199 articles were initially included. After removing duplicates, conference abstracts, studies that were not written in English, and studies where no full text was available, a total of 1199 articles were screened for eligibility based on their titles and abstract. Another 1127 studies were excluded, mainly because the studies either focused on malignancies other than lung cancer or used fluorescence imaging to evaluate molecular processes. Next, 72 full-text manuscripts were assessed, after which 29 articles were excluded. These articles either focused on other malignancies, only performed in vitro imaging, or used fluorescence imaging with fluorophores that have no excitation within the NIR spectrum. In total, 43 articles were included in this review. Figure 2 summarizes the selection of articles in a flowchart. Table 1 provides an overview of all fluorescent probes used for NIR fluorescence imaging of lung cancer and their optical properties.

### 3.2. Preclinical Studies

In total, 26 preclinical studies were included in this review. The results of these studies are summarized in Table 2.

#### 3.2.1. Non-Specific Fluorophores

In this review, three studies that examined non-specific fluorophores were included [36,37,38].

MHI-148 and IR-780 iodide are both heptamethine cyanine dyes that are absorbed and accumulated in cancer cells and not in healthy tissue due to the higher mitochondrial membrane potential in the tumor [39]. MHI-148 was tested in tumor-bearing mice [36]. Tumors showed fluorescence one hour after injection and reached a peak tumor-to-background ratio (TBR) of 3.62 after 24 h, which remained constant up to 72 h. The TBR is the ratio between the mean fluorescence intensity of the tumor and that of the surrounding tissue. To identify the tumor, a TBR of 1.5 is needed, but ideally, it should be above 2 to achieve more clear images. IR-780 was also tested in tumor-bearing mice [37]. Fourteen days after administration, a fluorescence signal was detectable in the tumor. Histopathological examination confirmed that IR-780 iodide was specifically accumulated in the tumor cells. Neither TBR nor signal-to-background ratio (SBR) was determined.

5-Aminolevulinic acid (5-ALA) is a naturally occurring amino acid that is involved in heme synthesis. 5-ALA induces the synthesis and accumulation of fluorescent protoporphyrin IX (PpIX) in various epithelia and neoplastic tissues and is mostly clinically applied in glioma patients for fluorescence-guided surgery [80]. 5-ALA was tested preclinically as a fluorescent agent in seven dogs with primary lung tumors [38]. Two to four hours prior to surgery, dogs were orally administered 20 mg/kg 5-ALA. The doses and time intervals were determined after a dose-escalation study in tumor-bearing mice. Six out of seven tumors were detectable with fluorescence imaging with a median in situ TBR of 2.1. Tumor margins were detectable in two dogs, while the remaining dogs had an unreliable fluorescence pattern. No additional lesions were found with fluorescence imaging or with pathological examination.

The optical properties of MHI-148 and IR-780 iodide are promising, as emission wavelengths around 800 nm are known to have less background signal than wavelengths around 700 nm. This is in line with the high TBR found in the MHI-148 study. However, the precise mechanism of these agents remains unclear. No in-human studies have yet been performed with either of these agents. An advantage of 5-ALA is that this agent is already approved by the FDA and EMA. However, 5-ALA fails to adequately detect tumor margins in lung cancer. This could possibly be explained by the higher background signal due to the optical properties of 5-ALA.

#### 3.2.2. Antibodies

Antibodies conjugated to fluorophores have dominated the field of tumor-targeted NIR fluorescence-guided surgery [81]. Since antibodies are already clinically available, they are relatively easy to implement as imaging agents in clinical settings. Three studies using antibodies conjugated to a NIR dye as a NIR fluorescent probe were included in this review [42,43,44].

Programmed death-ligand 1 (PD-L1) is a major ligand of programmed death 1 (PD-1), which is expressed on the surface of activated T cells. The binding of PD-L1 to PD-1 leads to the inhibition of T cells. This results in the inhibition of the anticancer immune response and cancer cell death [82]. PD-L1 is expressed in 50–75% of NSCLCs [83,84]. The PD-1/PD-L1 pathway is a target of currently available immunotherapy for lung cancer. The anti-PD-L1 antibody atezolizumab was conjugated to the NIR dye 800CW to create the fluorescent probe NIR-PD-L1-mAB [42]. The probe was tested in tumor-bearing mice with high PD-L1 expression. The tumor was detectable after 72 h and was best identified 120 h after administration. TBR and SBR were not reported. Specific fluorescence uptake in the tumor was higher compared to the control group of mice with low PD-L1 expression.

The monoclonal antibody mAb109 is a target for peroxiredoxin-I (Prdx I), which is part of an antioxidant protein family of six peroxiredoxins. Antioxidants play a role in protecting the lungs from free radicals and reactive oxygen species (ROS). However, a disbalance between ROS and antioxidant regulators can eventually lead to malignancies and also play a role in the behavior of tumor cells [85]. Prdx members are therefore linked to cell proliferation, differentiation, and apoptosis [86,87]. Prdx1 is overexpressed in 50–80% of all lung cancers [85,88]. To create a fluorescent probe, mAb109 was conjugated to the fluorophore cyanine 5.5 (Cy5.5) [43]. The probe was tested in tumor-bearing mice. Tumors were detectable with Cy5.5-mAb109 from 24 h to 16 days after administration. The highest TBR of 3.2 was reached after 24 h.

Humanized 173 (h173) is a monoclonal antibody that targets Axl, part of the tumor-associated macrophage (TAM) family, which consists of the receptor tyrosine kinases Tyro-3, Axl, and Mer. The TAM family plays a role in several cellular functions, such as natural killer cell differentiation, platelet aggregation, and macrophage clearance of apoptotic cells [89]. The expression of Axl is correlated with tumor invasion and progression and is overexpressed in 48–60% of NSCLCs [90,91]. The antibody h173 was conjugated to the NIR dye Cy5.5 and tested as a NIR fluorescent probe in tumor-bearing mice [44]. Tumors were best detectable with fluorescence imaging between 2 and 3 days after administration. Neither TBR nor SBR was determined. The fluorescence intensity was significantly higher compared to the control groups with either a non-targeted probe or Axl-negative tumors.

As described above, antibodies have been widely used for fluorescence imaging because of their clinical availability. However, of the three antibodies that were evaluated preclinically in the setting of NSCLC, only atezolizumab is currently available for use in patients. This, in combination with the favorable optical proportions of NIR-PD-L1-mAB, namely, the emission wavelength of 800 nm, makes this the most promising probe of these three.

#### 3.2.3. Nanoparticles

Nanoparticles are appealing targeting vehicles for fluorescence imaging since their surface can be chemically modified to improve the binding affinity of a probe to its target, increased in vivo stability can be achieved, and its pharmacokinetic properties can be optimized. The extravasation of nanoparticles into malignant cells is hypothesized to occur by passive diffusion through endothelial fenestrations of the leaking tumor vasculature. This phenomenon is known as the enhanced permeability and retention (EPR) effect. Nanoparticles can be conjugated to fluorophores and can also be combined with tumor-targeting moieties. In total, four studies using nanoparticles were included in this review [45,46,47,48].

The EGFR/MB-SHSi complex was produced by conjugating silica nanoparticles encapsulated with the fluorophore MB to anti-epidermal growth factor (EGFR) [45]. EGFR is overexpressed in 40–80% of NSCLCs and is a tyrosine kinase receptor associated with angiogenesis and a poor prognosis [92]. The probe was tested in tumor-bearing mice. The tumor was detectable after 1 h, and fluorescence intensities were strongest 6 h after administration. Neither TBR nor SBR was determined. Fluorescence signals were higher compared to the control group of mice receiving non-targeted MB-SHSi.

Glycol chitosan is a hydrophilic glycol group with a water-soluble chitosan derivative [93]. Glycol chitosan nanoparticles (CNPs) have been conjugated to the fluorophores Cy5.5 and ICG [46]. The probes Cy5.5-CNPs and ICG-CNPs were tested in tumor-bearing mice and rabbit models. Tumors were detectable up to 96 h after administration, and tumor margins were clearly visible during image-guided surgery in the rabbits. Neither TBR nor SBR was determined.

Calcium carbonate nanoparticles (CCPs) have low cytotoxicity and are highly biocompatible. CCPs entrapped with the fluorophore MB were conjugated to reconstituted high-density lipoproteins (rHDLs) [47]. A major component of HDL is apolipoprotein A-I, which has a high affinity to scavenger receptor class B type 1, which is expressed on most malignant cells [94]. The probe rHDLMB-CCPs was tested in tumor-bearing mice. Fluorescence signals were detectable at the tumor site after 2 h and were highest 6 h after administration. Neither TBR nor SBR was determined. Fluorescence intensities were higher compared to the control group, wherein mice received Lipos/MB-CCPs.

Superparamagnetic iron oxide nanoparticles (SPIONs) are nanoparticles with superparamagnetism under magnetic fields, biological compatibility, and high stability [95]. Polyethylene glycol (PEG) is often used to stabilize SPIONs. PEG-stabilized SPIONs were conjugated to folic acid (FA) [48]. FA targets the folate receptor alpha (FRα), which is expressed in 72–87% of lung adenocarcinomas and 13–57% of lung squamous cell carcinomas [96,97,98]. Tumors were detectable after administration of FA-PEG-SPIONs-Cy5.5. Neither TBR nor SBR was determined. Fluorescence signals in the lung were higher compared to the control group of mice that received untargeted PEG-SPIONs-Cy5.5.

Nanoparticles are promising targeting vehicles. However, the clinical translation of these nanoprobes is challenging. This is mainly because the biocompatibility and particularly the long-term toxicity of nanoprobes are not well established [99].

#### 3.2.4. Generated Probes

Besides the use of available targeted probes, there are also several techniques to generate specific targeted probes. Two studies using different techniques to generate a specific probe were included in this review [49,50].

Aptamers are chemical antibodies that can bind specifically to targets by folding into three-dimensional structures and therefore have a high binding affinity and specificity to their targets [100]. These aptamers are short oligonucleotides generated by the systematic evolution of ligands by exponential enrichment (SELEX), an in vitro selection method [101]. Four families of aptamers that bind to NSCLC adenocarcinomas were generated with SELEX. These aptamers are S1, S6, S11, and S15 [102]. S6 conjugated to the NIR dye cyanine 5 (Cy5) was tested in tumor-bearing mice [49]. Tumors showed fluorescence signals five minutes up to five hours after administration of Cy5-S6. The greatest signal was seen after three hours. Neither TBR nor SBR was determined. Control groups with either a negative control probe or a different tumor type showed limited to no fluorescence uptake at the tumor sites.

The peptide Pep-1, which specifically targets lung cancer, was found by phage display, which is a cell-specific peptide ligand selection method [103]. Pep-1 was conjugated to Cy5.5 and then tested in tumor-bearing mice [50]. On the 11th day after administration of Cy5.5-labeled Pep-1, high fluorescence signals were detectable in the tumor. Neither TBR nor SBR was determined. No to minimal fluorescence signals were seen in control groups with either a different phage, free Cy5.5, or another type of tumor.

Neither TBR nor SBR was reported in either of these studies. Since the emission wavelength of both probes is largely in the visible light spectrum, there is expected to be a relatively high background signal. In addition, the clinical translation of Pep-1 will be challenging because its counterpart protein has not been identified yet. Aptamers have been approved by the FDA in the past [104].

#### 3.2.5. Activatable Probes

Activatable probes are designed to be fluorescent after they have been turned on within the tumor or the tumor microenvironment. These probes are developed to reduce background signals. Activation occurs through a structural change within the fluorescent probe or by cleaving a quencher that was linked to the fluorophore. This activation of the probes can occur by enzymatic processes, chemical reactions with other analytes, or sensitivity to characteristics of the tumor microenvironment, such as hypoxia or a low pH [105]. In total, seven studies were included in this review that evaluated activatable probes for the use of fluorescence imaging of lung cancer [51,52,53,54,55,56,57].

The probe 6QCNIR is activated when it is processed by cysteine cathepsins [51]. Cysteine cathepsins are proteases that are highly upregulated in macrophages in the tumor microenvironment. These proteases promote tumor growth, angiogenesis, and invasion [106]. The fluorophore that is used in this probe is DyLight780-B1. The probe was tested in tumor-bearing mice. Tumors were detectable with fluorescence imaging with high contrast between tumor and normal tissues. Additional tumor lesions were detected. Histological examination showed that all fluorescent tissue was malignant. Neither TBR nor SBR was determined.

Prosense 680 and 750 are also probes activated by cathepsins [52]. Both probes were tested in tumor-bearing mice. The TBRs of the Prosense 680 and 750 groups were 9.3 and 8.5, respectively. TBR in the control group with mice that were not injected with fluorescent probes was around 1. Histological examination showed specific fluorescence uptake in the tumor center, but there were even higher fluorescence signals in the tumor periphery and immediately adjacent lung parenchyma. Lung parenchyma at a macroscopic distance from the tumor showed very low fluorescence.

A limitation of the use of protease-activated probes is the lack of selectivity. To overcome this, the AND-Gate probes were developed. AND-Gate probes must be processed by two tumor-specific enzymes to produce a fluorescence signal. Two AND-Gate probes were developed: DEATH-CAT 2 and FAP-CAT [53]. DEATH-CAT 2 is activated after processing by cysteine cathepsins and caspase 3, which is activated in apoptotic cells only. FAP-CAT is activated after processing by cysteine cathepsins and fibroblast activation protein α (FAP), which is found in invasive tumor borders. Both can be used to visualize lung carcinomas in mice with TBRs of 2.1 and 2.0, respectively. DEATH-CAT 2 was the best-performing probe in tumor-bearing mice. It was conjugated to the heptamethine cyanine fluorophore FNIR-tag for its application during robotic surgery in tumor-bearing mice. Tumors as small as 1 mm were visible during surgery. Hematoxylin and eosin (HE) staining showed that only malignant cells were fluorescent.

Another enzyme that is used to activate probes is calpain 2 (CAPN2). This is a protease activated by calcium and has a role in cell proliferation, cell migration, angiogenesis, and tumorigenesis [107]. The enzyme-activatable peptide probe HSA-CAPN2 was developed by conjugating a CAPN2 peptide sensor to human serum albumin (HSA), which serves as a carrier [54]. The CAPN2 peptide sensor consists of a CAPN2-specific peptide substrate, NIR dye cyanine 5 (Cy5), and the dark quencher BHQ-3. Tumors were detectable after two hours, and fluorescence signals in the tumor were the highest four hours after administration. Fluorescence imaging corresponded to bioluminescence images of implanted luciferase-labeled cells in the tumor. Neither TBR nor SBR was determined.

Q-cetuximab consists of an ATTO655 dye conjugated to cetuximab, which is an EGFR antibody [55]. ATTO655 can be quenched by the photo-induced electron transfer mechanism between the conjugated dye and tryptophan. The probe was tested in tumor-bearing mice. Eight hours after administration, the TBR was 4.28. Fluorescence imaging corresponded to bioluminescence images of implanted Lu2 cells in the tumor.

NIR-ASM consists of the NAD(P)H quinone oxidoreductase 1 (NQO1) substrate quinone propionic acid (QPA) conjugated to the fluorophore dicyanoisophorone (ASM) [56]. NQO1 is an antioxidant enzyme. As previously described, antioxidant enzymes protect cells from oxidative stresses caused by free radicals and ROS [108]. However, the reduction of certain quinones by NQO1 converts them into cytotoxic agents, which leads to cell death [109]. NQO1 is overexpressed in various types of cancers, such as lung cancer [110]. The non-fluorescent NIR-ASM will be activated when QPA, part of NIR-ASM, in the presence of NADH, undergoes a two-electron reduction by NQO1. The probe was tested in tumor-bearing mice. Fluorescence signals were only detectable in tumors and were the highest 30 min after injection. Neither TBR nor SBR was determined. In the control group of mice with NQO1-negative tumors, no fluorescence was seen.

Most cancer cells have extracellular acidosis due to the upregulation of aerobic glycolysis [111,112]. The pH-activatable aptamer probe (pH-AAP) consists of a cancer-specific aptamer molecule S6, fluorophore Cy5, and dark quencher BHQ2 [57]. Due to the low pH in tumor tissue, the quencher will be cleaved from Cy5. The probe will then be activated. The probe was tested in tumor-bearing mice. The tumors were detectable after 2 min, and fluorescence signals were the highest after 120 min. There were limited background signals. Neither TBR nor SBR was determined. Control groups with either a negative control probe or a different tumor type showed limited to no fluorescence uptake at the tumor sites.

Activatable probes can improve the speed of detection of tumors and the sensitivity and specificity of tumor detection compared to traditional “always-on” probes by reducing background signals, making them promising probes for future clinical use. However, the clinical potential of these probes for fluorescence-guided surgery in lung cancer has to be confirmed in clinical trials. Cathepsin-activated probes might give false-positive signals in inflammatory tissue since cathepsins are regulators of inflammatory processes [113].

#### 3.2.6. Integrin-Targeting Probes

Integrins are a family of cell adhesion receptors that have a crucial role in the development, progression, and metastatic potential of tumors. Integrins consist of α and β subunits [114]. Four articles that investigated integrin-targeting probes were included in this review [58,59,60,61].

The probe Cyp-GRD consists of the fluorophore cypate (cyp) and the inactive linear hexapeptide GRDSPK (GRD), which targets integrin αvβ3 [58]. The cyclic arginine-glycine-aspartate peptide antagonist (cRGDfK)-targeted probe consists of the fluorophore H3H and two copies of the arginine-glycine-aspartate (RGD) peptide, which binds to several integrins, including integrin αvβ3 [59]. The probe LXY30-biotin/streptavidin-Cy5.5 consists of the fluorophore Cy5.5 and LXY30, which targets integrin α3β1 [60]. The probe endostatin-Cy5.5 consist of the fluorophore Cy5.5 and endostatin, which binds to a variety of receptors, including vascular endothelial growth factor receptor 2 and 3 (VEGFR-2 and VEGFR-3) and integrin α5β1 and αVβ3 [61,115]. All four probes were tested in tumor-bearing mice. Tumor-specific fluorescence uptake was seen in all of these studies. The tumor-to-muscle ratio after administration of the cRGDfK probe was 3.6. Neither TBR nor SBR was determined in any of the other studies.

Most of these studies do not report a TBR or SBR, which makes it difficult to evaluate the efficacy of the probes and impossible to compare studies. Integrin-targeted probes have been shown to be able to detect colorectal cancer in patients during surgery [116].

#### 3.2.7. Others

Lastly, three articles were included that could not be accommodated in the above categories [62,63,64].

Hyaluronic acid is a polysaccharide that occurs naturally within the body and has interesting biochemical properties, such as non-immunogenicity, biocompatibility, and biodegradability. When applied to nanomaterials, hyaluronic acid has the potential for active targeting [117]. Hyaluronic acid has a strong binding affinity to CD44, a family of transmembrane proteins. CD44 is overexpressed in many cancer cells and is associated with the cell motility, invasive properties, and metastatic potential of tumors [118]. Hyaluronic acid nanogel (Hya-AT) was conjugated to the fluorophore Alexa Fluor 680 [62]. The probe was tested in tumor-bearing mice. Tumors were best detectable 8 h after administration. Neither TBR nor SBR was determined.

The delta-opioid receptor (δOR) is a transmembrane G-protein-coupled receptor that binds endogenous opioid peptides and opiates [119]. The endogenous opioid system is involved in tumorigenesis [120]. The δOR is overexpressed in lung cancer and absent in normal lung tissue [121]. A synthetic peptide antagonist (Dmt-Tic) of δOR was conjugated to the fluorophore 800CW [63]. The probe Dmt-Tic-IR800 was tested in tumor-bearing mice. Tumors were detectable with fluorescence imaging 24 h after injection. Neither TBR nor SBR was determined. Fluorescence signals were significantly higher compared to the control group of mice with δOR-negative tumors.

Retinoids are both natural and synthetic derivatives of vitamin A and are regulators of a variety of cellular functions. Retinoids are required for growth, vision, reproduction, immune function, hemostasis, and normal embryonic development [122]. In carcinogenesis, a common event is the deregulation of retinoid signaling [123]. Retinoic acid (RA) has been examined for the treatment and prevention of different types of cancer [124]. RA was conjugated to the fluorophore IRDye800CW [64]. The probe RA-IRDye800CW was tested in tumor-bearing mice. Tumors were best detectable 72 h after administration. The mean TBR was 2.15.

High doses of RA are associated with systemic toxicity. More research on the pharmacokinetics and biodistribution of Hya-AT and Dmt-Tic-IR800 is currently being performed. These three probes are all promising probes for the detection of lung cancer with NIR fluorescence imaging during surgery. However, the biocompatibility of the probes must be demonstrated, and their efficacy has to be confirmed in patient trials.

**Table 2:** Overview of results of preclinical studies.

Fluorescent Probe	Study Population	Doses	Time Interval	Control Group	Results	Toxicity	Ref
**Non-specific fluorophores**							
MHI-148	A549 mice (n = 5 per time interval)	1.5 nmol IV	1, 2, 6, 24, 48, 72, 96 and 120 h	Mice not injected with the probe (n = NR)	Tumors start showing fluorescence one hour after injection and reached a peak TBR of 3.62 after 24 h, which remains constant up to 72 h. Control mice did not show fluorescence at the tumor site.	Low cytotoxicity	[36]
IR-780 iodide	Chemically induced mouse models (n = NR)	0.2 mg/kg IV	14 d	NA	Tumors were detectable with fluorescence imaging. Histological examination confirmed that IR0780 iodide was specifically accumulated in the tumor cells.	NR	[37]
5-ALA	LKR or A549 mouse models (n = 3 per doses)	0, 10, 20, 40 or 100 mg/kg oral	1–8 h	NA	In the three highest doses groups fluorescence signals were detectable, with a comparable peak TBR (around 5) 1 hour after administration. The fluorescence intensities in the two highest groups were associated with higher TBRs at later time points.	Nausea	[38]
	Dogs with primary lung cancer (n = 7)	20 mg/kg oral	2–4 h		6 of 7 cancers were detectable with fluorescence imaging with a median TBR of 2.1. Tumor margins were detectable in two dogs, the others tumors had an unreliable fluorescence pattern. No additional lesions were found.		
**Antibodies**							
NIR-PD-L1-mAB	NCI-H2444 mouse models (PD-L1+) (n = 3–5)	22 μg IV	24, 48, 72, 96 and 120 h	NCI-H1155 (PD-L1 -) mouse models (n = 3–5)	The tumor was detectable after 72 h and was best identified after 120 h. Specific fluorescent uptake in the tumor was higher compared to the control group.	NR	[42]
Cy5.5 – mAb109	A549 mouse models (n = 4)	0.2 nmol IV	2, 24, 48 h and 5, 7, 9, 16 d	Mice injected with 0.5 nmol Cy5.5-ICG or co-injected with 500 μg unlabeled mAb109 (n = 4 per group)	Tumors were detectable from 24 h up to 16 days after the administration. The highest tumor to normal tissue ratio was reached after 24 h and was 3.2. Tumor to normal ratios were much lower in the control groups. Fluorescence imaging corresponded to bioluminescence images of co-implanted Luci cells in the tumor.	NR	[43]
h173-Cy5.5	A549 mouse models (n = 5)	30 μg IV	6 h, and 1, 2, 3, 4, 7 d	Mice injected with hIgG-Cy5.5 or mice with Axl negative tumors (H249 cells) injected with h173-Cy5.5 or hIgG-Cy5.5 (n = 5 per group)	Tumors were best detectable with fluorescence imaging 2–3 days after administration. The fluorescence intensity was significantly higher compared to the control groups.	NR	[44]
**Nanoparticles**							
Anti-EGFR/MB-SHSi	A549 mouse models (n = NR)	NR IV	1, 3, 6 h	Mice injected with non-targeted MB-SHSi (n = NR)	The tumor was detectable after 1 h and fluorescence intensities were strongest after 6 h. Fluorescence signals were higher compared to the control group.	Negligible cytotoxicity	[45]
Cy5.5-CNPs ICG-CNPs	VX2 mouse models (n = 3)	22.5 mg/kg IV	Up to 96 h	NA	The tumors in the Cy5.5-CNPs group were detectable after 24 h and maximum fluorescence intensities were measured in the tumor between 48 and 96 h. Tumors in the ICG-CNPs group were detectable for up to 96 h.	No cytotoxicity	[46]
ICG-CNPs	VX2 rabbit models (n = 3)	22.5 mg/kg IV	Up to 96 h	Rabbits injected with 2 mg/kg free ICG	Tumors were detectable with fluorescence imaging up to 96 h after injection, with maximum fluorescence intensities after 48 h. Tumor margins were clearly visible. In the control group only 30 min after injection a minimal fluorescence signal was detectable.	No cytotoxicity	[46]
rHDL/MB-CCPs	A549 mouse models (n = NR)	NR IV	2, 4, and 6 h	Mice injected with lipos/MB-CCPs (n = NR)	The tumor was detectable after 2 h and fluorescence signals were highest after 6 h. Fluorescence intensities were higher and tumor targeting was more specific compared to the control group	No significant cytotoxicity	[47]
FA–PEG– SPIONs–Cy5.5	Urethane mouse models (n = 5)	5 mg/kg IV	6 and 24 h	Mice injected with non-targeted PEG–SPIONs–Cy5.5 (n = 5)	Fluorescence signals in the lung were higher compared to the control group at both time intervals.	Cy5.5 in high concentration is cytotoxic.	[48]
**Generated probes**							
Cy5-S6	A549 mouse models (n = NR)	0.5 nmol IV	5 min and 3, 5 h	Mice injected with control probe Cy5-Lib and mice contralateral injected with Tca8113 tongue carcinoma (n = NR)	Tumors showed fluorescence after five minutes to five hours after the administration. The greatest signal was seen three hours after the injection. Control groups showed limited to no fluorescence uptake at the tumor sites.	NR	[49]
Cy5.5-labeled Pep-1	A549 mouse models (n = 3)	NR IV	1–11 d	Mice receiving a different phage, free Cy5.5 or mice with different tumor types receiving Cy5.5-labeled Pep-1 (n = 3 per group)	On the 11th-day, high fluorescence signals were detectable in the tumor. Histological examination showed that Pep-1 was strongly detected in tumor tissue. No to minimal fluorescent signals were seen in control groups	NR	[50]
**Activatable probes**							
6QCNIR	KPT mice infected with Lenti-Cre virus (n = NR)	20 nmol IV	6 h	Healthy mice injected with the probe (n = NR)	Tumors were detectable with high contrast between tumor and normal tissue. Additional tumor lesions were detectable. Histological examination showed that all fluorescent tissue was malignant. Control mice showed only background fluorescence.	NR	[51]
Prosense 680 Prosense 750	LLC mouse models (n = 5 per probe)	2 nmol IV	24 h	Mice not injected with the probe (n = 5)	TBRs of the Prosense 680 and 750 groups were 9.3 and 8.5, respectively. Histological examination showed specific fluorescence uptake in the tumor center, but even higher fluorescence signals in the tumor periphery and immediately adjacent lung parenchyma to this. Lung parenchyma at macroscopic distance from the tumor showed very low fluorescence. TBR in the control was around 1.	Probes appear to be safe	[52]
DEATH-CAT-FNIR	krasG12D/+p53−/− adenocarcinoma mouse models (n = NR)	20 nmol IV	16 h	NA	Tumors as small as 1 mm were visible with fluorescence imaging. Histological examination showed that only malignant cells showed fluorescence.	NR	[53]
HSA-CAPN2	A549 mouse models (n = 5)	125 μg IV	1, 2, 4, 9, and 18 h	Mice injected with HSA–Cy5, CAPN2, or HSA-CAPN2 after IT injection of ALLN, a CAPN2 inhibitor (n = 5 per group)	Tumors were detectable after two hours and fluorescence signals in the tumor were the highest four hours after administration of the probe. Fluorescence signals were significantly higher than in all three control groups. Fluorescence imaging corresponded to bioluminescence images of co-implanted Luc cells in the tumor.	NR	[54]
Q-cetuximab	A549 mouse models (n = 3)	50 μg IV	8 h	Mice injected with 50 μg ON-Cetuximab or 100 μL PBS (n = 3 per group)	The TBRs in the Q-cetuximab, ON-Cetuximab, and PBS groups were 4.28 ± 1.13, 1.48 ± 0.06, and 1.14 ± 0.08, respectively. Fluorescence imaging corresponded to bioluminescence images of co-implanted Lu2 cells in the tumor.	NR	[55]
NIR-ASM	A549 mouse models (n = NR)	5 mg/kg IV	5, 10, 20, 30 min	Mice were contralaterally injected with MDA-MB-231 cells (NQO1 negative) (n = NR)	Fluorescence signals were detectable in tumors in both mouse models and were the highest 30 min after injection. No fluorescence signals were detectable in NQO1 negative tumors or healthy mice.	No toxicity	[56]
	LLC mouse models (n = NR)			Healthy mice injected with the probe (n = NR)			
pH-AAP	A549 mouse models (n = NR)	0.24 nmol IT	2 to 300 min	A549 tumor-bearing mice injected with pH-ALP or SMMC-7721 tumor-bearing mice injected with pH-AAP (n = NR)	The tumors were detectable after 2 min and fluorescence signals were the highest after 120 min. There was a limited background signal. The new probe, pH-AAP, showed less background signal as compared with the previous probe Cy5-S6. Control groups with either a negative control probe or different tumor type showed limited to no fluorescence uptake at the tumor sites.	NR	[57]
**Integrin-targeting probes**							
Cyp-GRD	A549 mouse models (n = NR)	0.3 μmol/kg IV	2, 8, and 24 h	Mice injected with linear or cyclic RGD conjugated to cypate (n = NR)	Tumor-specific fluorescence uptake was seen 8 h after injection and the tumor was best detectable with fluorescence imaging after 24 h. The fluorescence signals were higher compared to the control groups.	No cytotoxicity	[58]
cRGDfK-targeted probe	A549 mouse models (n = 8)	5 nmol IV	90 and 180 min	Mice injected with an untargeted probe (n = 8)	The tumors were detectable at both time intervals with comparable fluorescence intensities. The average tumor-to-muscle ratio was 3.6. In the control group, there was only minimal fluorescence visible at the tumor site.	Low phototoxicity	[59]
LXY30	H3255 and PDX human lung squamous cell carcinoma mouse models (n = NR)	1.8 nmol IV	6 h	Mice injected with untargeted biotin/streptavidin-Cy5.5 complex (n = NR)	Fluorescence signals were detectable in the tumor in both H3255 and PDX mice. Histopathological examination showed that the probe was specifically taken up in the tumor. Fluorescence signals were higher as compared with the control group.	NR	[60]
Endostatin-Cy5.5	LLC mouse models (n = 3)	20 mg/kg IV or IP	1–168 h	Mice injected with alone endostatin or Cy.5.5 and mice not injected at all (n = 3 per group)	Tumors were detectable with fluorescence imaging from 18 to 114 h after intraper injection. The maximum fluorescence intensity was reached after 42 h. After intraveintravenous injection, fluorescence intensity reached the maximum after 3 h and fluorescence signals were detectable up to 72 h. Control groups did not show tumor-specific fluorescence uptake.	NR	[61]
**Others**							
HyA-AT-Alexa680	A549 mouse models (n = 5)	5 mg/kg IV	5 min and 1, 8, 24, and 48 h	Mice injected with native Hya – Alexa680 (n = 5)	Till 1 hour after injection, high background signals were detected. After 8 h the tumor was best detectable. The residence time in the tumor was shorter in the control group.	Cytotoxic at incubation time of 72 h and 1 mg/ml nanogel concentration	[62]
Dmt-Tic-IR800	DMS-53 (δOR+) mouse models (n = 4)	40 nmol/kg IV	24 h	Mice were contralaterally injected with H1299 (δOR-) cells	The tumors were detectable with fluorescence imaging and had significantly more fluorescent uptake compared to the δOR negative control tumors.	NR	[63]
RA-IRDye800CW	A549 mouse models (n = 10)	10 nmol IV	1–9 d	NA	The highest fluorescent signals were detected after 72 h. The TBR was 2.15.	Cytotoxicity at doses higher than saturation dose 0.1/mg/kg/week	[64]

*Abbreviations*: NR—not reported; NA—not applicable; IV—intravenous; TBR—tumor-to-background ratio; NIR—near-infrared; PD-L1—programmed death-ligand 1; mAb—monoclonal antibody; Cy5.5—cyanine 5.5; ICG—indocyanine green; EGFR—epidermal growth factor; MB—methylene blue; SHS—superhydrophobic silica; CNPs—glycol chitosan nanoparticles; rHDL—high-density lipoprotein; CCPs—calcium carbonate nanoparticles; FA—folic acid; PEG—polyethylene glycol; SPIONs—superparamagnetic iron oxide nanoparticles; Cy5—cyanine 5; HSA—human serum albumin; CAPN2—calpain 2; ASM—dicyanoisophorone; pH-AAP—pH-activatable aptamer probe; LLC—Lewis lung carcinoma; KPT—KrasLSL-G12D/+; p53f lox/flox; R26LSL-tdTomato/+; PBS—phosphate-buffered saline; IT—intratumorally; cyp(ate)—bis-propanoic acid cyanine dye; GRD—linear hexapeptide GRDSPK; H3H—dimeric bis(hydroxyphenyl); PDX—patient-derived xenograft; IP—intraperitoneally; HyA-AT—Hyaluronic acid nanogel; δOR—delta-opioid receptor; RA—retinoic acid; 5-ALA—5-aminolevulinic acid.

### 3.3. Clinical Studies

#### 3.3.1. ICG

ICG is a non-specific fluorophore that binds to plasma proteins and lipoproteins because of its amphiphilic and water-soluble anionic properties. When administered systemically, ICG binds to plasma proteins (albumin) and transfers from the plasma to the hepatic parenchymal cells, and it is cleared via the biliary system [125]. The peak absorption of ICG is 780 nm and, after binding to plasma proteins, emits light at 805 nm [126]. ICG was described as a fluorescent agent in lung cancer [65,66,67,68,69,70,71]. One of these studies is a preclinical trial, four are clinical trials, and two are a combination of both. An overview of the results of these studies is shown in Table 3.

A dose-escalation study in a tumor-bearing mouse model showed that the optimal timing for ICG administration for intraoperative imaging was 24 h prior to surgery and the optimal dose was 5 mg/kg [65]. These results were confirmed in patients [65,66]. In all human studies combined, 94 patients with 134 pulmonary nodules were included. NIR fluorescence-guided surgery was able to detect 109 lesions (81.3%). In total, 14 additional lesions were detected, of which 9 were malignant. False-negative and false-positive percentages in situ were 9.1–13% and 11.8–18.2%, respectively. The in situ sensitivity was 86.4–89.3% [66,67,68,69,70]. False-positive lesions were seen in inflammation and obstructive pneumonia [69,70]. A study in both dogs and humans showed that detection of adequate resection margins was not possible when the tumor was surrounded by inflammation or atelectasis [67].

In all of these studies, ICG was administered intravenously; however, the possibility of intraoperative imaging using ICG inhalation has also been studied in a preclinical setting [71]. Fluorescence should be visible only in healthy lung tissue and not in tumor tissue because the tumor causes airflow obstruction and the destruction of alveoli. A proof-of-principle study in tumor-bearing mouse models showed that fluorescence imaging after ICG inhalation was able to visualize adequate tumor margins, as confirmed with histological examination. Tumor margins were visible 10 min to 24 h after inhalation of ICG via nebulization, and the peak signal was reached after 1 h. Inhalation of ICG had a significantly higher signal-to-noise ratio in mice than an intravenous injection of ICG. A dose-escalation study in tumor-bearing rabbits showed that tumor margins were visible from a dose of 0.25 mg/kg, while a higher dose resulted in higher fluorescence intensity in normal tissue. One rabbit showed a false-positive lesion, which turned out to be atelectasis. The use of ICG inhalation was tested ex vivo in human lung specimens. The resected lung lobes of 6 patients were inserted with an endotracheal tube, and ICG was delivered into the lobes via nebulization. The resection margins were clearly visible in all six lung specimens, with a mean tumor margin detection efficiency (SBR in tumor/SBR in healthy tissue) of 2.9.

Both intravenous injection and inhalation of ICG were able to distinguish tumor from healthy tissue; however, both methods give false-positive signals in atelectasis and/or inflammation [67,71]. Relatively high doses of ICG were used (up to 5 mg/kg in clinical trials). No side effects were seen in any of these studies [65,66,67]. There was no significant correlation found between the size of the nodules or the maximum standardized uptake values (SUVs) on preoperative PET/CT and the fluorescence intensity of the nodules [68,70]. Fluorescence imaging was able to detect tumors from a size of 2 mm [68,71]. However, a significant correlation was seen between the fluorescence intensity and the depth of the tumor [68]. NIR fluorescence imaging with ICG enabled the visualization of tumors up to 13–14 mm from the surface, as observed with preoperative CT imaging [68,69,70]. There was observer bias in all of these studies because there is no quantitative method for assessing fluorescence signals. Another limitation of these studies is the small sample size.

#### 3.3.2. OTL38

OTL38 is a folate analog ligand conjugated to S0456, an indole-cyanine green-like dye. OTL38 excites at a maximum wavelength of 774–776 nm and has a peak emission of 794–796 nm [127]. OTL38 targets the folate receptor alpha (FRα), which is expressed in 72–87% of lung adenocarcinomas and 13–57% of lung squamous cell carcinomas [96,97,98]. Eight studies were found that analyzed OTL38 as a fluorescent agent in lung cancer, of which six were clinical trials, and two were preclinical studies combined with a proof-of-concept study in patients. An overview of the results of these studies is shown in Table 4.

A dose-escalation study was performed in tumor-bearing mice xenografts [73]. The highest TBR of 3.04 was seen at a dose of 0.025 mg/kg and 24 h after iv administration. This was then evaluated as a proof of principle in patients with pulmonary nodules. The time interval chosen for this study was 3 to 6 h prior to surgery. This different time interval compared to the optimal time interval in mice was chosen for two reasons. Firstly, according to previous studies with folate receptor-targeted agents, the pharmacokinetics seems to be faster in humans than in mice. Secondly, patients do not need to come to the hospital on another day for the infusion, as the drug can now be given on the day of surgery.

**Figure 3:**
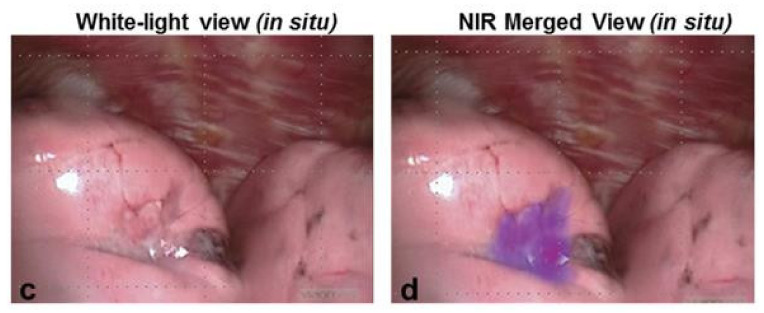
Fluorescence imaging results using OTL38. *A squamous cell lung cancer was clearly visible (TBR 3.7) with intraoperative fluorescence imaging after intravenous injection of 0.025 mg/kg OTL38 6 h prior to surgery* [76]. *It shows an image in white light (**c**) and a merged image of white light and near-infrared (**d**)*.

In all studies combined, 486 patients with 505 pulmonary nodules were included. Of all lesions, 53.8–100% were fluorescent during surgery, and the mean in situ TBR was 2.4–3.8 (Figure 3). In total, 40 additional lesions were detected, of which 25 were malignant. The in situ false-negative and false-positive percentages were 0–38.5% and 0–12.9%, respectively. Most false-positive lesions were granulomas, and most false-negative lesions were localized deeper in the lung, outside the detection limit of NIR light. The sensitivity and specificity were 58.3–100% and 20–42.9%, respectively. The positive and negative predictive values were 87.1–94.2% and 26.7–50%, respectively [72,73,74,75,76,77,78,79]. All of these values apply to in situ results and will be higher when fluorescence assessment is performed ex vivo since TBRs of the lesions are higher when measured in back table pathology assessments.

A large retrospective cohort study showed that there was no significant difference between the TBR of malignant and benign lesions [78]. The sensitivity in patients with adenocarcinoma (84%) was higher than in patients with squamous cell carcinoma (58.3%) [73,76]. This is in line with the difference in FRα expression between these two histological cancer types. In three studies, fluorescence imaging was used to examine the wound bed to check for tumor-positive resection margins. Fluorescence signals in the wound bed were found in 10–17.4% of the subjects. Histopathological examination showed that 56.3–100% of tumor margins were true positives [72,77,79]. Clinically significant events, which means that the surgical plan is changed by the results of fluorescence imaging, happened in 0–45% of patients [72,73,74,75,76,77,78,79].

There was no significant correlation found between fluorescence signals and the SUVmax on PET/CT or with the size or location of the lesion in different lobes. However, there was a significant correlation between fluorescence signals and the depth of the lesion [72,75,76,78]. The deepest lesion found was located 20 mm below the surface of the lung, as observed on the preoperative CT scan. Fluorescence imaging was able to detect lesions from a size of 4 mm [72]. Adverse events were described in four studies. In two of these studies, no adverse events were seen [72,78]. In the other two studies, 15–21.8% of the patients did have adverse events, most of which were complaints of nausea. One patient had desaturation, which resolved after stopping the infusion. Another patient had chest pain, while the electrocardiogram (ECG) and troponins were normal [73,77]. There was observer bias in all of these studies due to the lack of a quantitative method for assessing fluorescence signals. Another limitation of most of these studies is the small sample size.

### 3.4. Metastases

Two studies were found that performed NIR fluorescence-guided surgery in patients with lung cancer metastases. Both studies used 5-ALA as an imaging agent in patients with cerebral metastases, including metastases from lung cancer. Knipps et al. included 11 patients with cerebral metastases of lung cancer [41]. Three hours prior to surgery, patients were orally administered 20 mg/kg 5-ALA. Only 3 out of the 11 metastases were detectable with fluorescence imaging. Charalampaki et al. included five patients with cerebral metastases of lung cancer [40]. Patients were orally administered 20 mg/kg 5-ALA, three hours prior to surgery. All tumors showed fluorescence signals. One metastasis showed only fluorescence uptake in the capsule, and in one metastasis, the fluorescence pattern was homogeneous. All metastases were completely resected, as confirmed by MRI. Both studies mainly included patients with adenocarcinoma.

## 4. Future Perspectives

### 4.1. Clinical Trials

As described above, multiple fluorescent probes have been tested preclinically in animals, but only a few have subsequently been tested in patients. Translating preclinical in vivo diagnostics to clinical settings is challenging due to several factors, including acceptance of small effect sizes, intellectual property rights, unpredictable industry timetables, fear of litigation, complex regulatory requirements, and the high costs of drug development [128]. Currently, multiple clinical trials are underway. Two antibody-conjugated fluorescent probes that are being tested in patients with lung cancer in these trials are nimotuzumab-IRDye800CW and panitumumab-IRDye800CW [129,130]. These probes both target EGFR. The studies are expected to finish inclusion in 2022 and 2023, respectively. Another study in which patients with different tumor types, including NSCLC, are included is investigating ONM-100 [131]. This is a pH-activatable fluorescent agent consisting of a pH-sensitive amphiphilic polymer conjugated to ICG, which activates in the acidic extracellular tumor environment [132]. This study was estimated to be completed by the end of 2021. Furthermore, SGM-101, an antibody against carcinoembryonic antigen coupled with the fluorochrome BM104, is currently being investigated in patients with pulmonary nodules in a multicenter pilot study [133]. The end of the study is expected to be in 2022.

### 4.2. Photoacoustic Imaging

NIR light has a higher tissue penetration compared to visible light, but nevertheless, the inability to visualize structures deeper than approximately 10 mm below the surface remains an important limitation. The maximum depth of tumor detection in the lung is greater than the previously described 10 mm in other solid tumors such as the liver; however, the depth in the lung is determined based on a preoperative CT scan, and because the lung is collapsed during surgery, the actual penetration depth of NIR light in the lung is lower than the 20 mm described in this review. A new molecular imaging technique that is under investigation, which has a greater tissue penetration depth, up to 7 cm, is photoacoustic imaging [134]. Photoacoustic imaging is based on the photoacoustic effect, which relies on the principle that absorbed light is converted into heat and generates a temperature rise. This will lead to thermoelastic expansion, which results in the emission of acoustic waves. These acoustic waves can be detected by using ultrasonic transducers [135,136]. Photoacoustic imaging thus combines the contrast of optical imaging with the high spatial resolution and penetration depth of ultrasound imaging. Photoacoustic imaging distinguishes between various tissues because the difference in light absorption rates between various tissues causes different acoustic waves [137]. A preclinical study was performed to investigate the use of photoacoustic imaging to localize pulmonary nodules. This study suggests that photoacoustic imaging with ICG as an imaging agent may be useful for the identification of pulmonary nodules localized deeper in the tissue [138].

### 4.3. NIR-II Window

The second near-infrared bioimaging window (NIR-II) ranges from 1.000 to 1.700 nm. Due to less photon scattering and absorption in tissue, NIR-II imaging has a deeper imaging depth compared to NIR-I imaging and has less interference from background noise, known as autofluorescence [139,140]. This was confirmed in multiple studies in which NIR-II imaging had higher SBRs than NIR-I imaging [141,142]. NIR-II imaging seems able to overcome the two biggest limitations of NIR-I imaging. However, there are still some barriers to overcome before NIR-II imaging can be implemented in clinical settings. First, most of the current NIR-II fluorescent molecules have poor physiological stability and are hydrophobic, and in addition, some of these molecules are inorganic fluorophores that have high toxicity, are slowly metabolized, and do not target specific tissues [139,143]. Secondly, various molecular interactions lead to quenching, causing a reduction in emitted light from fluorescent molecules [144]. Thirdly, only a few NIR-II camera systems are currently available, and the development of these cameras is very expensive. Most importantly, only preclinical NIR-II fluorescent probes are available at the moment. Translation of preclinical research to clinical settings is a long process; thus, clinical application may still be far away.

### 4.4. Quantification

In most clinical applications to date, surgeons must interpret the fluorescence signal themselves, causing interobserver variability. To overcome this, it is necessary to quantify the fluorescence signal. However, no standardized methods to quantify fluorescence signals are currently available. Designing and validating these methods is challenging because numerous factors influence the fluorescence signal, such as absorption, scattering, camera distance and angulation, and autofluorescence [19]. Most clinical studies use the semi-quantification parameter TBR or SBR. Several studies have been performed to evaluate the quantification of NIR fluorescence imaging of colorectal perfusion using ICG to prevent anastomotic leakage [145,146]. These studies showed that quantitative analysis of ICG perfusion can be used to detect colorectal segments with poor perfusion. A recent review showed that inflow parameters have a better association with clinical endpoints compared to fluorescence intensity parameters [147]. These studies all investigated the quantification of NIR fluorescence imaging of tissue perfusion. At the moment, no data are available on the quantification of tumor fluorescence signals. More research on the quantification of fluorescence signals is necessary before this technique can be expected to generate reliable, reproducible results in clinical practice.

### 4.5. Combination with Targeted Drug Delivery

A recent development is the combination of targeted drug delivery with fluorescence imaging. Targeted drug delivery systems consist of an antitumor drug combined with a cancer-cell-specific ligand. Since drugs are specifically delivered to cancer cells, while most healthy tissue is unaffected, this system could lead to improved antitumor efficacy and reduced toxicity. Combining targeted drug delivery systems with a fluorescent agent could create a theranostic platform that would lead to effective antitumor therapy along with real-time noninvasive imaging of the therapeutic response [148,149]. The tyrosine kinase inhibitor gefitinib and p53 gene therapy have been linked to a NIR dye to use this platform in lung cancer [150,151]. These probes were successfully studied in tumor-bearing mouse models. Both probes specifically accumulated in the tumor, where the drugs and NIR dyes were successfully delivered. A limitation of the clinical use of NIR fluorescent agents in this technique is the limited depth penetration.

### 4.6. Image-Guided Surgery Combined with Photodynamic Therapy

Photodynamic therapy (PDT) is a treatment that uses a light-sensitive medicine and a light source to destroy premalignant and malignant cells. Patients are treated with a photosensitizer that will be activated by light of a specific wavelength, creating reactive oxygen species that kill nearby cells [152]. Since photosensitizers are fluorescent, they can also be used as agents in fluorescent image-guided surgery. Using PDT as adjuvant therapy after image-guided surgery to destroy missed cancer cells and/or unresectable tissues could reduce cancer recurrence [153]. Several preclinical studies have been performed to evaluate theranostic probes for the combination of fluorescence imaging and PDT in lung cancer. Hyaluronic acid, matrix metalloproteinase MMP2 polypeptide, and PD-L1 peptides have been used as tumor targets, and chlorin e6 and IR 780 iodide have been used as photosensitizers. Different types of nanoparticles have been used as drug delivery systems [154,155,156,157,158].

## 5. Conclusions

NIR fluorescence-guided surgery has the potential to identify lung cancers during surgery, detect additional lesions, and prevent tumor-positive resection margins. Two fluorescent agents, ICG and OTL38, have been tested in patients with lung cancer. Both agents have been shown to visualize the tumor during surgery, but they cannot effectively distinguish between malignant and benign tissue, particularly in inflammation or atelectasis. Many fluorescent agents have been developed and tested in a preclinical setting; however, little in-human data are available. More research, especially in a clinical setting, has to be performed before NIR fluorescence-guided surgery can be advanced as the standard of care for tumor targeting in patients with lung cancer.

## Figures and Tables

**Table 1:** Overview of all fluorescent probes used for the detection of lung cancer and their optical properties.

Fluorescent Probe	Molecular Target	Fluorophore	Peak Excitation Wavelength	Peak Emission Wavelength	Ref.
Preclinical tested probes					
Non-specific fluorophores					
MHI-148	NA	MHI-148	760	800	[36]
IR-780 iodide	NA	IR-780 iodide	780	799	[37]
5-ALA	NA	PpIX	405/633	635/710	[38,40,41]
Antibodies					
NIR-PD-L1-mAb	PD-L1	800CW	780	800	[42]
Cy5.5–mAb109	Prdx I	Cy5.5	675	694	[43]
h173-Cy5.5	Axl	Cy5.5	675	694	[44]
Nanoparticles					
Anti-EGFR/MB-SHSi	EGFR	MB	665	686	[45]
Cy5.5-CNPs	NA	Cy5.5	675	694	[46]
ICG-CNPs	NA	ICG	780	805	[46]
rHDL/MB-CCPs	SR-B1	MB	665	686	[47]
FA-PEG-SPIONs-Cy5.5	FA	Cy5.5	675	694	[48]
Generated probes					
Cy5-S6	S6	Cy5	649	666	[49]
Cy5.5-labeled Pep-1	Pep-1 (CAKATCPAC)	Cy5.5	675	694	[50]
Activatable probes					
6QCNIR	Cathepsins	DyLight780-B1	783	799	[51]
Prosense 680	Cathepsins	Prosense 680	680	700	[52]
Prosense 750	Cathepsins	Prosense 750	650	780	[52]
DEATH-CAT-FNIR	Cathepsins + caspase 3	Heptamethine cyanine	765	788	[53]
HSA-CAPN2	CAPN2	Cy5	649	666	[54]
Q-cetuximab	EGFR	ATTO655	600	684	[55]
NIR-ASM	NQO1	ASM	460	646	[56]
pH-AAP	S6	Cy5	649	666	[57]
Integrin-targeting probe					
Cyp-GRD	α_V_β_3_	Cypate	780	830	[58]
cRGDfK-targeted probe	Integrins	H3H	644	668	[59]
LXY30-biotin/streptavidin-Cy5.5	α3β1	Cy5.5	675	694	[60]
Endostatin-Cy5.5	VEGFR-2, VEGFR-3, α5β1, αVβ3	Cy5.5	675	694	[61]
Others					
HyA-AT-Alexa680	CD44	Alexa fluor 680	679	702	[62]
Dmt-Tic-IR800	δOR	800CW	780	800	[63]
RA-IRDye800CW	Retinoids	800CW	780	800	[64]
Clinical tested probes					
ICG	NA	ICG	780	805	[65,66,67,68,69,70,71]
OTL38	FRα	S0456	774–776	794–796	[72,73,74,75,76,77,78,79]

*Abbreviations*: NA—not applicable; 5-ALA—5-aminolevulinic acid; PpIX—protoporphyrin IX; NIR—near-infrared; PD-L1—programmed death-ligand 1; mAb—monoclonal antibody; Cy5.5—cyanine 5.5; EGFR—epidermal growth factor receptor; MB—methylene blue; SHS—superhydrophobic silica; CNPs glycol—chitosan nanoparticle; ICG—indocyanine green; rHDL—reconstituted high-density lipoprotein; CCPs—calcium carbonate nanoparticles; SR-B1—scavenger receptor class B type 1; FA—folic acid; PEG—polyethylene glycol; SPIONs—superparamagnetic iron oxide nanoparticles; Cy5—cyanine 5; HSA—human serum albumin; CAPN2—calpain 2; ASM—dicyanoisophorone; pH-AAP—pH-activatable aptamer probe; cyp(ate)—bis-propanoic acid cyanine dye; GRD—linear hexapeptide GRDSPK; H3H—dimeric bis(hydroxyphenyl); RGD tripeptide—Arg-Gly-Asp; VEGFR-2—vascular endothelial growth factor receptor 2; VEGFR-3—vascular endothelial growth factor 3; HyA-AT—hyaluronic acid nanogel; δOR—delta-opioid receptor; RA—retinoic acid.

**Table 3:** Overview of study results analyzing the use of ICG as a fluorescent agent for the detection of lung cancer.

Author and Year	Study Population	#Patients (Lesions)	Doses	Timing	Highlights
Jiang 2015	LLC mouse models	25 (25)	0.71, 2.0, 5.0, 7.5, 10.0 mg/kg IV	1 min–72 h	The optimal doses and timing in mice were 5 mg/kg and 24 h. This was confirmed in patients. TBRs in the 5 mg/kg group were 3.1–3.7.
	Patients with pulmonary nodules	6 (6)	0.71, 2.0, 5.0 mg/kg IV	1 day	
Newton 2018	Patients with NSCLC	18 (18)	1–3 mg/kg IV (*n* = 9), 4–5 mg/kg IV	1 day	In the 4–5 mg/kg group, 8 of 9 tumors were fluorescent, with a mean TBR of 2.70. In the 1–3 mg/kg group, 1 of 9 tumors were fluorescent, with a mean TBR of 1.49.
Holt 2014	Dogs with primary lung cancer	8 (8)	5 mg/kg IV	1 day	All tumors were fluorescent with a mean in situ SBR of 8.8. NIR imaging was able to detect adequate resection margins in 5 tumors. The other 3 tumors had peritumoral inflammation.
	Patients with pulmonary nodules	5 (5)			All nodules were fluorescent, with a mean SBR of 8.1. In 4 nodules, NIR imaging was able to detect adequate resection margins, and none of these nodules showed inflammation. The other tumor was surrounded by atelectasis.
Okusanya 2014	Patients with pulmonary nodules	18 (18)	5 mg/kg IV	1 day	Of 18 lesions, 14 showed fluorescence in situ. There were 5 additional nodules detected. All fluorescent nodules were malignant, and the mean SBR was 2.2. Of non-fluorescent lesions, 3 of 4 were malignant. The sensitivity was 86.4%.
Mao 2017	Patients with pulmonary nodules	36 (76)	5 mg/kg IV	1 day	Of 76 lesions, 68 were detected with IGS, of which 63 lesions were malignant. All 8 non-fluorescent lesions were malignant. The mean SBR was 3.29. In total, 9 additional lesions were identified. Of these, 5 were false positives. The sensitivity was 89.3%.
Kim 2016	Patients with pulmonary cancer	11 (11)	1 mg/kg IV	1 day	Of 11 lesions, 10 were fluorescent, of which 8 lesions were malignant. The two false-positive lesions had a pathological complete response after neoadjuvant therapy and new obstructive pneumonia. The non-fluorescent lesion was a false negative. The sensitivity was 88.9%.
Quan 2020	LLC mouse models	32 (32)	1.0 mg/kg inhalation	10 min–24 h	The tumor margin could be visualized by fluorescence imaging, as confirmed with histological examination. Tumor margins were visible 10 min to 24 h after inhalation of ICG, with a signal peak after 1 h. Inhalation of ICG had a significantly higher tumor margin detection efficiency as compared with intravenous injection of ICG.
	VX2 rabbit models	20 (20)	0.1, 0.25, 0.5, 1.0 mg/kg inhalation		Tumor margins were visible from a dose of 0.25 mg/kg, and higher doses resulted in higher fluorescence intensity in normal tissue. One rabbit showed a false-positive lesion, which turned out to be atelectasis.
	Human lung specimen	6 (6)	NR inhalation	NR	All tumor margins were visible, with a mean tumor margin detection efficiency (ratio between signal-to-noise ratio in tumor tissue and that in healthy tissue) of 2.9.

*Abbreviations:* LLC—Lewis lung carcinoma; ICG—indocyanine green; IV—intravenous; NSCLC—non-small cell lung carcinoma; NR—not reported; TBR—tumor-to-background ratio; SBR—signal-to-background ratio.

**Table 4:** Overview of study results analyzing the use of OTL38 as a fluorescent probe for the detection of lung cancer.

Author and Year	Study Population	#Patients (Lesions)	Doses	Timing	TP (%)	FP (%)	TN (%)	FN (%)	#Additional Lesions (%TP)	Sensitivity (%)	Specificity (%)	PPV (%)	NPV (%)
Predina 2018	A549 mice models	15 (15)	0.0, 0.0125, 0.025, 0.050 and 0.25 mg/kg IV	Up to 8 days	NR	NR	NR	NR	NR	NR	NR	NR	NR
	Patients with pulmonary nodules	10 (10)	0.025 mg/kg IV	3–6 h	8 (80)	0 (0)	1 (10)	1 (10)	3 (100)	84.6	*	*	*
Keating 2017	Dogs with primary lung cancer	10 (10)	0.185 mg/kg IV	2–3 h	10 (100)	*	*	0 (0)	NR	100	*	*	*
	Patients with adenocarcinoma	3 (3)	0.025 mg/kg IV		3 (100)	*	*	0 (0)	NR	100	*	*	*
Azari 2021	Patients with pulmonary nodules	279 (279)	0.025 mg/kg IV	Mean 7.7 h	217 (77.8)	32 (12.9)	8 (2.9)	22 (7.9)	NR	90.8	20	87.1	26.7
Gangadharan 2021	Patients with pulmonary nodules	92 (92)	0.025 mg/kg IV	2–6 h	NR	NR	NR	NR	24 (37.5)	NR	NR	NR	NR
Predina (2) 2018	Patients with ground-glass opacity	20 (21)	0.025 mg/kg IV	3–6 h	15 (71.4)	1 (4.8)	0 (0)	5 (23.8)	NR	75	*	*	*
Predina (3) 2018	Patients with squamous cell cancer	12 (13)	0.025 mg/kg IV	3–6 h	7 (53.8)	*	*	5 (38.5)	NR	58.3	*	*	*
Predina (4) 2018	Patients with adenocarcinoma	20 (21)	0.025 mg/kg IV	3–6 h	17 (81)	*	*	4 (19)	4 (100)	84	*	*	*
Predina 2017	Patients with pulmonary nodules	50 (66)	0.025 mg/kg IV	3–6 h	56 (84.6)	4 (6.1)	3 (4.5)	3 (4.5)	9 (100)	95.6	42.9	94.2	50

*Abbreviations:* TP—true positive; FP—false positive; TN—true negative; FN—false negative; PPV—positive predictive value; NPV—negative predictive value; NR—not reported. * Value cannot be determined, since only patients with proven malignancies were included or there were less than 5 benign and/or malignant lesions, and therefore, a representative value cannot be determined.

## Appendix A. Search Strategy per Database

PubMed:

(“Lung Neoplasms”[MeSH Terms] OR “lung neoplasm*”[Title/Abstract] OR “lung cancer*”[Title/Abstract] OR “pulmonary neoplasm*”[Title/Abstract] OR “pulmonary cancer*”[Title/Abstract] OR “lung tumor*”[Title/Abstract] OR “lung tumour*”[Title/Abstract] OR “lung nodule*”[Title/Abstract] OR “pulmonary nodule*”[Title/Abstract]) AND (“Fluorescence imaging”[Title/Abstract] OR ((“spectroscopy, near infrared”[MeSH Terms] OR “near-infrared”[Title/Abstract] OR “near-infra-red”[Title/Abstract] OR “NIR”[Title/Abstract]) AND “imaging”[Text Word]) OR ((“Optical Imaging”[MeSH Terms:noexp] OR “Optical Imaging”[Title/Abstract]) AND (“Fluorescence”[MeSH Terms] OR “fluorescen*”[Title/Abstract])) OR ((“intraoperative”[Text Word] OR “intra-operative”[Text Word]) AND “imaging”[Text Word] AND (“Fluorescence”[MeSH Terms] OR “fluorescen*”[Title/Abstract]))).

Cochrane:

(“lung neoplasm*”:ti,ab,kw OR “lung cancer*”:ti,ab,kw OR “pulmonary neoplasm*”:ti,ab,kw OR “pulmonary cancer*”:ti,ab,kw OR “lung tumor*”:ti,ab,kw OR “lung tumour*”:ti,ab,kw OR “lung nodule*”:ti,ab,kw OR “pulmonary nodule*”:ti,ab,kw) AND (((“near-infrared”:ti,ab,kw OR “near-infra-red”:ti,ab,kw OR ‘NIR’:ti,ab,kw) AND ‘imaging’:ti,ab,kw) OR (“fluorescen*”:ti,ab,kw AND “optical imaging”:ti,ab,kw) OR ((‘intraoperative’:ti,ab,kw OR ‘intra-operative’:ti,ab,kw) AND ‘imaging’:ti,ab,kw AND “fluorescen*”:ti,ab,kw) OR (“fluorescence imaging”:ti,ab,kw)).

Web of Science:

(“lung neoplasm*” OR “lung cancer*” OR “pulmonary neoplasm*” OR “pulmonary cancer*” OR “lung tumor*” OR “lung tumour*” OR “lung nodule*” OR “pulmonary nodule*”) AND (((“near-infrared” OR “near-infra-red” OR ‘NIR’) AND ‘imaging’) OR (“fluorescen*” AND “optical imaging”) OR ((‘intraoperative’ OR ‘intra-operative’) AND ‘imaging’ AND “fluorescen*”) OR (“fluorescence imaging”)).

Embase:

(exp lung tumor/ or “lung neoplasm*”.ti,ab. or “lung cancer*”.ti,ab. or “pulmonary neoplasm*”.ti,ab. or “pulmonary cancer*”.ti,ab. or “lung tumor*”.ti,ab. or “lung tumour*”.ti,ab. or “lung nodule*”.ti,ab. or “pulmonary nodule*”.ti,ab.) AND ((exp fluorescence imaging/ or “fluorescence imaging”.ti,ab.) OR ((exp near infrared spectroscopy/ or “near-infrared”.ti,ab. or “near-infra-red”.ti,ab. or “NIR”.ti,ab.) AND “imaging”.mp.) OR (“Optical Imaging”.ti,ab. AND (exp fluorescence/ or “fluorescen*”.ti,ab.)) OR (((“intraoperative” or “intra-operative”).ti,ab.) AND (exp fluorescence imaging/ or “fluorescence imaging”.ti,ab.) AND (exp fluorescence/ or “fluorescen*”.ti,ab.)).
